# Treatment Strategy for Posterior Malleolar Fractures: Different Operative Strategies Are Needed for Each Morphological Type

**DOI:** 10.3390/jcm14041216

**Published:** 2025-02-12

**Authors:** Byung-Ki Cho, Sivakumar Allur Subramanian, Jihyun Hwang, Collin Lee, Young Phil Yune, Sung Jae Kim, Seung Myung Choi

**Affiliations:** 1 Department of Orthopaedic Surgery, College of Medicine, Chungbuk National University, Cheongju 28644, Republic of Korea; titanick25@naver.com; 2 Department of Orthopedic Surgery, Hallym University Dongtan Sacred Heart Hospital, Hwaseong 18450, Republic of Korea; drsivaphdbio@gmail.com; 3 Department of Biomedical Engineering, Johns Hopkins University School of Medicine, Baltimore, MD 21205, USA; jhwang50@jh.edu; 4 Department of Biology, University of Maryland—College Park, College Park, MD 20742, USA; clee1241@terpmail.umd.edu; 5 Department of Orthopaedic Surgery, Daejeon Bon Hospital, 114, Gyeryong-ro, Yuseong-gu, Daejeon 34188, Republic of Korea; yuneyp@naver.com

**Keywords:** posterior malleolus, morphology, classification, operative treatment, trauma, syndesmosis stabilization, posttraumatic osteoarthritis

## Abstract

**Background:** The operative indication for posterior malleolar fracture (PMF) remains controversial. This study aimed to assess the midterm outcomes of PMF treatment for developing a treatment strategy for each morphological type. **Methods:** In this retrospective analysis, patients undergoing operative treatment for an unstable ankle fracture involving PMF were included after at least 3 years of follow-up. PMFs were classified by fracture morphology according to the Haraguchi classification. This study divided the entire cohort into three independent populations based on the types of PMF. For each population, patients were further categorized into two groups depending on whether PMF was surgically fixed or not, and comparisons were made between these two groups. Demographic data, functional and radiographical outcomes were compared between two groups in each of the three populations. **Results:** With a total of 472 patients, the mean patient age was 45.8 years, and the mean follow-up was 51 months. For type 1 fracture, a total of 237 cases were found. Quality of reduction by CT (QRC) was mostly good in both groups (83.6% vs. 83.3% in the non-fixation vs. fixation group, respectively, *p* = 0.269). Functional and radiological outcomes between both groups showed no significant difference. For type 2 PMFs, a total of 199 cases were found, and QRC was significantly different between the two groups (good grade, 5.4% vs. 60.7% in the non-fixation vs. fixation group, respectively, *p* < 0.001). The radiological and clinical outcomes of the PMF fixation group were statistically superior to those of the non-fixation group (both *p* < 0.001). For type 3 fractures, a total of 36 cases were found. In all the cases in this group, surgical fixation of PMF was not performed. Only the syndesmosis instability was analyzed as a viable factor to be considered for achieving favorable surgical outcomes. PMF fixation group showed significantly more postoperative complications (24.4% vs. 40.4%, non-fixation vs. fixation, respectively, *p* < 0.001). Major complications in the fixation group were deep wound infection (6.8%), superficial peroneal nerve injury (6.8%), and hallux flexion deficit (5.0%). **Conclusions:** Different treatment strategies seem to be required for each PMF morphological subtype. Further studies with more detailed designs for each PMFs are warranted for more clinically related results that are helpful for making practical surgical decisions.

## 1. Introduction

Posterior malleolar fractures (PMFs) can occur in up to 40% of ankle fractures and have been associated with the development of post-traumatic osteoarthritis (PTOA) [1,2,3,4,5]. Classically, the goal of operative PMF treatment has been to restore articular congruity based on biomechanical evidence demonstrating decreased joint contact area with increasing PMF fragment size, thereby increasing load concentration. The biomechanical importance of posterior malleolar fractures lies in their critical role in maintaining ankle joint stability, as well as posterior talofibular and syndesmotic stability. Inadequate treatment of posterior malleolar fractures can lead to improper load distribution across the tibial plafond, disrupt normal joint mechanics, and potentially result in post-traumatic arthritis [6,7,8]. Therefore, the decisive factor for operative treatment has long been the articular fragment’s size and displacement; the critical size was 25–33% of the articular surface and fragment displacement was >2 mm [6,9,10]. However, the clinical implication of this decreased joint contact area remains unknown [11,12]; furthermore, recent biomechanical evidence has shown that the posterior malleolus plays a minor role in the ankle joint’s stability and contact stresses [6,11,13].

With knowledge of the three-dimensional PMF pathoanatomy, attempts have been made to classify PMF according to its morphology in relation to the mechanism of injury using computed tomography (CT) [1,6,14,15,16,17]. PMF morphology is increasingly recognized as having greater clinical relevance than the size of the fracture fragments involving the articular surface. The traditional emphasis on fragment size has been challenged, particularly the description that fragments involving less than 25% of the articular surface can be managed non-operatively. Research indicates that even smaller fragments, depending on their morphology, can contribute to ankle instability and warrant surgical intervention [1,11,14,15,16,18,19,20,21,22,23,24]. Accordingly, several authors have developed a protocol for morphology-based PMF treatment; however, the operative indications, techniques, and approaches for this injury remain controversial [6,12,17,18,19].

The current concept of PMF fixation has shifted from indirect and percutaneous anterior-to-posterior screws to direct reduction and plate fixation through a posterior approach for restoring articular congruity and fibular length and stabilizing syndesmosis. Approaches such as posterolateral and posteromedial surgical techniques have been developed to facilitate direct visualization and fixation of these fractures. Studies have reported improved functional outcomes with these methods, emphasizing the importance of addressing the posterior fragment in ankle fracture management. [11,12,25,26,27,28,29]. However, there have been concerns regarding this technique being potentially associated with an increased incidence of postoperative complications, including metalwork irritation, deficit hallux flexion, and foot numbness, possibly resulting in inferior functional outcomes compared with that of nonoperative treatment [11,13,21].

Therefore, this study aimed to assess the midterm radiological and clinical outcomes of the treatment of unstable ankle fractures with posterior malleolar involvement to develop a treatment strategy for each PMF type based on the classification system according to fracture morphology. We hypothesized that the PMF treatment strategy could be stratified according to fragment morphology related to fracture pathoanatomy and that the trend of routine plate fixation should be adopted with caution.

## 2. Materials and Methods

### 2.1. Demographic Data

**Figure 1:**
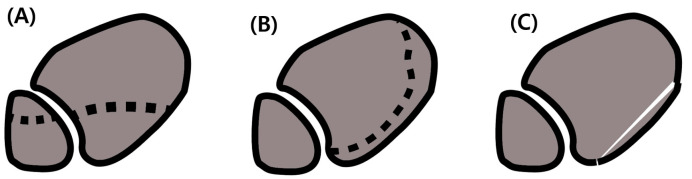
Schematic description of Haraguchi’s classification for posterior malleolar fracture, (**A**) Type 1 fracture, posterolateral oblique fracture (dotted line; fracture line), (**B**) Type 2 fracture, medial extension type, fracture line to the anterior part of the medial malleolus, (**C**) Type 3 fracture, the small-shell type, cortical shell-shaped fragments at the posterior cortical lip [15].

Using a prospective database and following institutional review board approval, we retrospectively reviewed patients who underwent operative treatment for unstable ankle fractures with posterior malleolar involvement from January 2014 to December 2017. For subjects’ inclusion in the current study, we used retrospective hospital electronic medical charts review and radiographic evaluation data, including pre-and postoperative simple radiographs, postoperative weight-bearing radiographs, and CT scanning. The patients were classified by PMF morphology according to Haraguchi’s classification [15] (Figure 1), and the outcomes of the group in which PMF was fixed and of that where PMF was unfixed were compared (PMF fixation and non-fixation groups, respectively).

**Figure 2:**
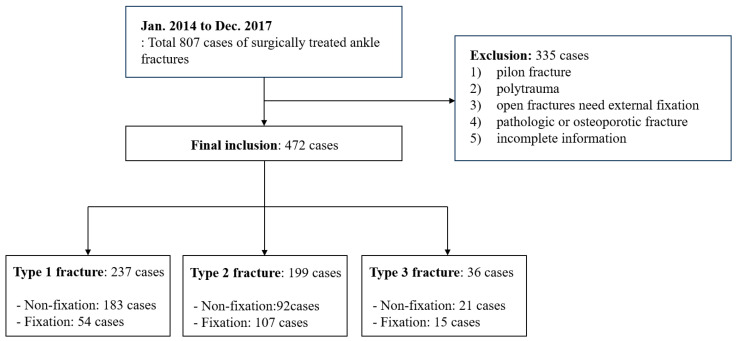
Among a total of 807 cases, 472 cases were included in the final analysis. Three types of fracture groups were independently analyzed for comparative analysis based on surgical intervention.

The inclusion criteria were (1) age >18 years and <70 years; (2) trimalleolar or equivalent fractures representing Arbeitsgemeinschaft für Osteosynthesefragen AO/Orthopaedic Trauma Association (AO/OTA) type 44B or C fractures, including Weber type B or C injury [30]; and (3) at least 3 years of follow-up. Exclusion criteria were (1) posterior pilon fractures (those with the posterior fragment extending into the anterior colliculus of the medial malleolus or comprising 50% of the incisura fibularis tibiae [12]; (2) polytraumas; (3) open fractures or those that underwent a staged protocol with temporary external fixation; (4) pathological fractures including osteoporotic fracture (bone mineral density T-score < −2.5) and Charcot arthropathy; and (5) incomplete information. The design of the current study is depicted in Figure 2.

### 2.2. Radiographic and Clinical Assessment

Patients underwent radiographic and clinical assessments preoperatively, at 6 weeks, 3, 6, and 12 months postoperatively and annually thereafter. The preoperative radiographic assessment included fracture configuration and injury severity. Non-weight-bearing anteroposterior, lateral, and mortise plain radiographs, as well as 3D-reconstructed CTs, were used to classify the fractures according to the Lauge-Hansen system and Weber’s classification. PMFs were classified by fracture morphology according to Haraguchi’s classification based on CT [15], in which two fellowship-trained foot-and-ankle specialists independently evaluated the fractures in a blinded manner. Disagreement was resolved by a third independent fellowship-trained trauma surgeon.

The postoperative radiographic assessment included the reduction quality, residual intra-articular step-off at the posterior tibial plafond, and PTOA development. Non-weight-bearing radiography and immediate postoperative CT were employed to assess the reduction quality and residual step-off. The reduction quality was determined based on the McLennan–Ungersma method [31]. Good reduction was defined as restoration of the fibular length, <2 mm posterior displacement, and <1 mm increase in medial clear space. Fair reduction was defined as <2 mm fibular shortening, 2–4 mm posterior displacement, 2 mm lateral displacement, and 1–3 mm increase in medial clear space. Poor reduction was defined as >2 mm fibular shortening, >4 mm posterior displacement, >2 mm lateral displacement, and >3 mm increase in medial clear space. The posterior residual step-off was considered a ≥1 mm displacement perpendicular to the articular surface of the posterior tibial plafond evaluated by the CT sagittal plane with the largest measurable diameter [14,28,32]. The weight-bearing radiographs, including anteroposterior and lateral ankle views, and the hindfoot alignment radiograph were obtained at 3 months postoperatively to evaluate PTOA development.

Clinical assessments were performed with the American Orthopaedic Foot and Ankle Society (AOFAS) hindfoot score [33], Olerud and Molander score (OMAS) [34], Foot and Ankle Outcome Score (FAOS), visual analog scale (VAS), and complications. Postoperative complications included superficial/deep wound infection, nerve injury, posterior metalwork discomfort, or the need for revision procedures. Superficial wound infections were defined as infections at operative sites treatable with conservative management, including oral antibiotics and dressings, without the need for readmission or intervention. Deep wound infections were defined as infections that require readmission or further interventions, including intravenous antibiotics, metalwork removal, and debridement.

### 2.3. Operative Technique

Surgeries for the current study were done by two surgeons with clinical experiences of five to 10 years who have worked in the same institute during the study period. The PMF treatment strategy was categorized into two groups: (1) posterior malleolus fixed by direct reduction and plate or screw fixation through a posterior approach and (2) unfixed posterior malleolus. The PMF approach was decided at the surgeon’s discretion. For the fixation group, fixation was performed with patients in the prone position before lateral and medial malleolar fracture fixation [17,26,27,32,35]. In type 1 PMF, the posterolateral approach for simultaneously accessing the distal fibular fracture and PMF was generally used [12,17,26]. Subcutaneously, the sural nerve or short saphenous vein superficial to the investing fascia is at risk and should be identified and protected [21,29,36]. In type 2 PMF, since the posteromedial fragment was located below the posterolateral fragment and extended to the anteromedial direction, the posteromedial fragment was fixed before the posterolateral fragment. Otherwise, there is a risk of medial translation of the posteromedial fragment due to the overcompression of the posterolateral fragment [17,26]: if necessary, a separate posteromedial incision in addition to the posterolateral approach was required to fully access the posteromedial fragment, depending on the fracture morphology. Options for internal fixation include using one or two 3.5-mm partially threaded cortical screws with or without washers or a posterior buttress plate.

For the non-fixation group, the patients were treated in the supine position. For both groups, after fixation of the lateral and medial malleolar fractures, stress examinations were performed for potential syndesmotic stabilization. Syndesmotic instability was defined as any widening of the distance between the medial edge of the fibula and incisura fibularis tibiae or medial clear space through either the external rotation or hook test detected during the operation [18,32,35,37]. When syndesmotic instability was detected, the distal tibiofibular joint was reduced using a reduction clamp in the neutral axis and fixed using a trans-syndesmotic screw or suture button. The lateral and medial malleolus fractures were fixed according to the AO/OTA operative principle, which did not differ between the groups. The deltoid ligament was repaired when a medial clear space >4 mm or any lateral tibial shift on intraoperative stress test following the lateral malleolar fracture and syndesmosis injury (if observed) was stabilized: a medial longitudinal incision was made, and the deltoid ligament was fixed with one or two anchor sutures or absorbable material.

In the case of posterior dislocation, unacceptable reduction of the posterior tibial plafond or syndesmotic instability after fixation of the lateral/medial malleolar fractures among patients who initially underwent the operation in the supine position without PMF fixation, the PMF was additionally fixed in the prone position.

We regarded achieving good or fair reduction as an acceptable reduction, as confirmed by intraoperative fluoroscopy, and all the patients in this study had an acceptable reduction and proper metalwork placement [38,39].

All patients were followed up according to the standard postoperative rehabilitation protocol, and all patients were immobilized for the first 1–2 weeks postoperatively with a short leg brace, followed by a boot with active and passive range of movement exercises or cast at the treating surgeon’s discretion with non-weight-bearing for 4–6 weeks. Weight-bearing activity was allowed when radiographical evidence of healing was combined with clinical examination findings.

### 2.4. Statistical Analysis

The total study cohort was divided into three populations based on the Haraguchi classification of posterior malleolar fractures (PMFs). Within each population, patients were further categorized into two groups: a non-fixation group and a surgical fixation group, and comparisons were made between these two groups. Comparisons between the populations were not performed as part of the primary analysis; however, for the analysis of complications, all populations were combined to compare the complication profiles between the non-fixation and surgical fixation groups.

Data are presented as mean and standard deviation. Data normality was verified using the Kolmogorov–Smirnov test. The respective PMF types and the fixation and non-fixation groups were compared using the Student’s t-test or Pearson’s chi-squared test. Significance levels for all analyses were set at *p* < 0.05. All statistical analyses were performed with SPSS software version 23.0 (IBM Corp., Armonk, NY, USA).

## 3. Results

A total of 472 patients were included in the current study. At surgery, the mean patient age was 45.8 ± 14.1 (range, 18–70) years, and the mean follow-up was 51 (range, 36–120) months. The demographic characteristics included age at the time of operation, sex, injury mechanism, and body mass index. A total of 237 (50.2%), 199 (42.2%), and 36 (7.6%) patients had fracture types 1, 2, and 3, respectively. PMF fixation was performed in 22.8%, 53.8%, and 0% of the patients with fracture types 1, 2, and 3, respectively (*p* < 0.001), and 161 (34.1%) and 311 (65.9%) patients were included in the fixation and non-fixation groups, respectively.

During the operation, nine patients initially treated in the supine position required additional posterior malleolar fixation in the prone position: two for posterior dislocation, three for syndesmotic instability, and four for unacceptable reduction of the posterior malleolar fragment after fixation of the lateral/medial malleolar fractures and syndesmotic stabilization. All these nine patients had type 2 PMF and were assigned to the PMF fixation group.

### 3.1. Comparison Between the Fixation and Non-Fixation Groups Within Type 1 Fracture

**Figure 3:**
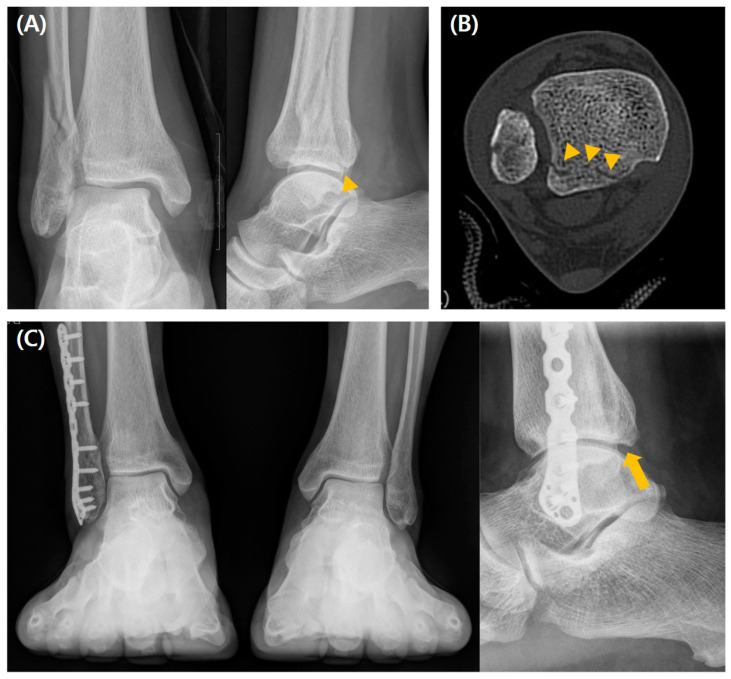
A case of a 56-year-old man who underwent conservative management for type 1 PMF. (**A**) Preoperative radiographs show a fracture line (arrowhead), and (**B**) preoperative CT scan images show a posterior malleolar fragment representing a type 1 fracture line (arrowheads) without the ICF. (**C**) Weight-bearing radiographs obtained 3 years postoperatively show acceptable reduction and bone healing (arrow).

The intercalary fragment (ICF) assessed by preoperative CT was more frequently observed in the fixation group (*p* < 0.001). There were no significant postoperative intergroup differences in reduction quality assessed by immediate postoperative CT, residual step-off, or PTOA development. Moreover, there were no significant intergroup differences in the clinical values, including mean AOFAS, OMAS, FAOS, or VAS scores at the final follow-up. Postoperative complications occurred more frequently in the fixation group (37% vs. 18.6%, *p* = 0.008) (Table 1). A radiographic case example of a type 1 fracture is depicted in Figure 3.

### 3.2. Comparison Between the Fixation and Non-Fixation Groups Within Type 2 Fracture

**Figure 4:**
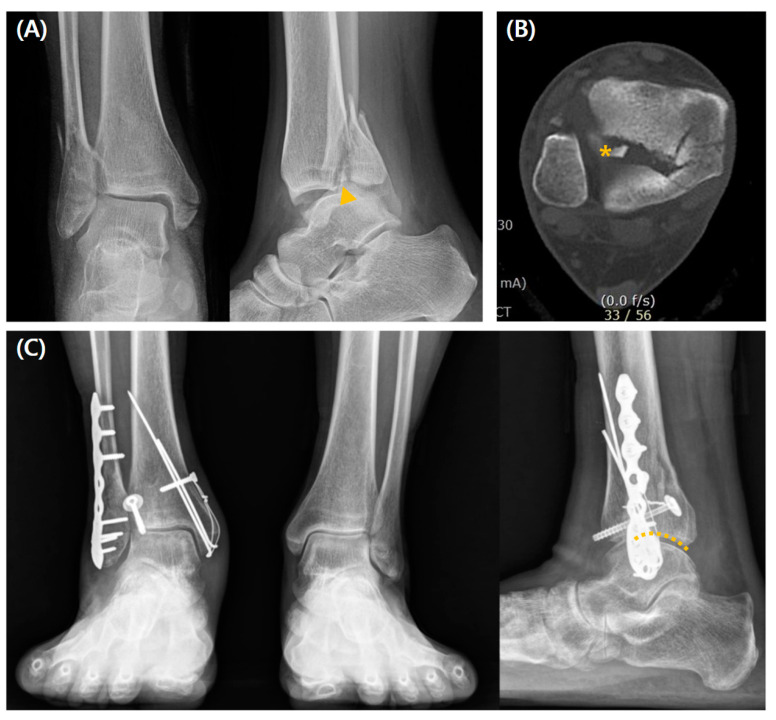
A case of a 44-year-old woman who underwent a direct approach and posterior screw fixation for type 2 PMF. (**A**) Preoperative radiographs show comminuted fracture (arrow), and (**B**) preoperative CT scan images show a posterior malleolar fragment representing type 2 fracture with the ICF (asterisk) and step-off. (**C**) Weight-bearing radiographs obtained 3 years postoperatively show good reduction without the development of PTOA (dotted line; anatomically reduced joint surface).

The rates of poor reduction assessed by the CT, postoperative step-off, and PTOA development were significantly higher in the non-fixation group (all, *p* < 0.001). All the measured clinical values were statistically superior for the fixation group (*p* < 0.001). Although not statistically significant, there was a trend toward a higher complication rate in the fixation group (42.1% vs. 34.8%, *p* = 0.366) (Table 2). A radiographic case example of a type 2 fracture is depicted in Figure 4.

A logistic regression analysis was performed on variables showing significant differences between the surgical fixation and non-fixation groups in Table 3 to analyze the factors influencing the decision for surgical fixation of posterior malleolar fractures that predict favorable treatment outcomes. The dependent variable was defined based on an AOFAS score threshold of 90. Independent variables included in the regression model were those that demonstrated statistically significant differences according to the surgical fixation status of the posterior malleolar fracture. These variables comprised quality of reduction by CT, step-off, and PTOA. Additionally, basic demographic data, including age, sex, and BMI, were incorporated as independent variables. Among the postoperative factors determined by the surgical fixation status of posterior malleolar fractures, PTOA [odds ratio = 6.212 (1.275 to 30.270), *p* = 0.024] and step-off [18.945 (3.942 to 91.044), *p* < 0.001] were observed to influence functional treatment outcomes (Table 3). This suggests that for type 2 posterior malleolar fractures, where significant preoperative articular displacement is observed, considering surgical fixation may contribute to predicting favorable treatment outcomes.

### 3.3. Comparison Between the Syndesmotic Stabilization and Nonstabilization Groups Within Type 3 Fracture

**Figure 5:**
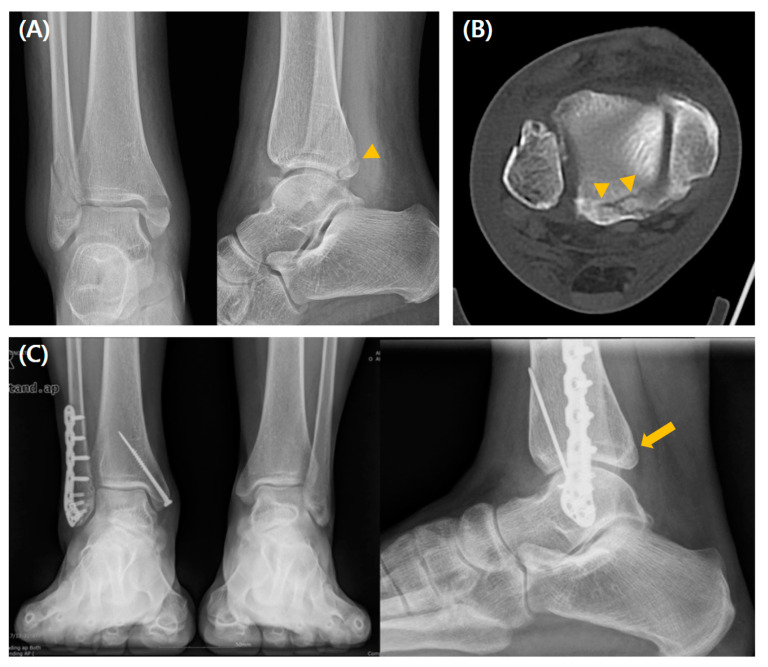
A case of a 34-year-old man who underwent conservative management without syndesmotic stabilization for type 3 PMF. (**A**) Preoperative radiographs show a small avulsion-type posterior lip fracture (arrowhead), and (**B**) preoperative CT scan images show a posterior malleolar fragment representing a type 3 fracture (arrowhead). (**C**) Weight-bearing radiographs obtained 2 years postoperatively show good reduction and bone healing (arrow).

All patients with type 3 fractures underwent conservative PMF management; for concomitant syndesmotic instability, they underwent syndesmotic stabilization. At the final follow-up, there were no significant intergroup differences in radiological and clinical parameters, including the postoperative complications, with the numbers available (Table 4). A radiographic case example of a type 3 fracture is depicted in Figure 5.

### 3.4. Comparison of Posterior Screw Fixation Versus Plate Fixation

The plate fixation proportion was larger for patients with type 2 PMF than for patients with type 1 PMF (85.2% vs. 62.7%, *p* < 0.042). Although the ICFs were more frequently observed in the plate group than in the screw group (*p* = 0.01), there was a trend toward a lower rate of poor reduction quality (0% vs. 6.7%, *p* = 0.093) and PTOA development in the plate group than in the screw group (7.4% vs. 14.2%, *p* = 0.522). The clinical values and postoperative complication rate did not differ significantly between the groups (Table 5).

### 3.5. Complications

Overall, postoperative complications occurred more frequently in the fixation group than in the non-fixation group (40.4% vs. 24.4%, *p* < 0.001). Notably, deep wound infections and limitation of plantar flexion on the hallux occurred more frequently in the fixation group (*p* < 0.001). When comparing plate fixation with screw fixation, posterior metalwork irritation and hallux flexion deficit is seen to occur more frequently in the plate group, which resolved with conservative care (*p* < 0.001) (Table 6 and Table 7).

### 3.6. Power Analysis

A post hoc power analysis was conducted using Pearson’s chi-square test to examine whether the sample size was sufficient to detect a significant difference between the fixation and non-fixation groups in PTOA development at the final follow-up for each PMF type. For type 1 PMF, a noninferiority verification study was performed to determine whether conservative management was not inferior to PMF fixation: at least 46 and 156 patients would be required for the fixation and non-fixation groups, respectively, with 95% power at 5% significance and a noninferiority margin of 0.1. Our study sample (54 and 183 patients for the fixation and non-fixation groups, respectively) was, therefore, adequate. For type 2 PMF, a superiority test for the finding that PTOA was more frequent in the non-fixation group was performed: at least 36 and 33 patients would be required for the fixation and non-fixation groups, respectively, with 95% power at 5% significance. Our study sample (107 and 92 patients for the fixation and non-fixation groups, respectively) was, therefore, also adequate. For type 3 PMF, the power analysis could not be performed because all patients in this group were treated with conservative management.

## 4. Discussion

The indication for PMF fixation or its optimal method has not been fully established [6,12,17,18,19,26]. Several studies have attempted to establish treatment strategies according to the respective morphological PMF subtypes; however, a generalizable conclusion could not be drawn due to their relatively small samples and short follow-ups [6,11,26]. We assessed the radiological and clinical outcomes of various modalities for the treatment of each morphological PMF type with a relatively large sample and a mean follow-up of >50 months. Our study is expected to provide additional information on PMF characteristics and establish a strategy for treating this challenging injury.

In type 1 PMF, ICFs were significantly more frequent in the fixation group than in the non-fixation group. ICF might prevent the reduction of the posterior plafond and is considered a prognostic factor for PTOA development; thus, it could be addressed operatively [6,12,22]. Sultan et al. [22] suggested that ICFs < 2 mm in diameter should be considered removed, and proper reduction should be performed for those >2 mm through the posterolateral or posteromedial approach. Given the higher rate of postoperative complications in the fixation group, efforts should be made to reduce the risk of postoperative complications related to the operative procedure. Although our results showed a trend toward a higher rate of poor reduction quality and PTOA in the non-fixation group than in the fixation group, no significant intergroup differences were observed in the radiological and functional outcomes during a follow-up of >3 years. There might be situations in which the operative approach is eminently indicated, such as an ICF possibly preventing the reduction of the posterior malleolus or posterior subluxation of the talus even after syndesmotic stabilization in addition to lateral/medial malleolar reduction [13,22,35]. Except for these situations, the non-fixation method combining appropriate syndesmotic fixation might show results comparable to the operative approach for type 1 PMF.

In type 2 PMF, more favorable radiological and clinical outcomes were obtained by the fixation group than the non-fixation group. Our findings agreed with previous studies that demonstrated the efficiency of the direct approach and internal fixation in terms of articular congruity restoration and improved functional outcomes. This type of PMF that involves the anterior portion of the medial malleolus usually shows comminuted fragments and more ICF that warrant surgical reduction and fixation [6,12,26,27,29,32,35,40,41,42]. However, considering the higher rate of hallux flexion deficit or metalwork irritation in plate osteosynthesis, posterior screw osteosynthesis could be considered for better clinical results [11,21]. There are few studies comparing the direct reduction and screw-versus-plate fixation of PMF. In a prospective comparative study of posterior screw and plate fixations through the posterolateral approach with a follow-up of approximately 3 years, Erdem et al. [43] obtained satisfactory and equivalent radiological and functional outcomes for both groups.

The type 3 PMF mechanism has been considered a bony avulsion of the posterior inferior tibiofibular ligament (PITFL) caused by talus external rotation [16,17,19,44]. Therefore, it can be hypothesized that syndesmotic fixation restores ankle joint stability. According to our results, however, only 15 of the 36 patients with type 3 PMF required syndesmotic stabilization, which did not affect the radiological and clinical results. This is possible because the PITFL contributes to only 42% of the stability that the four component ligaments of the syndesmosis provide [45], and the syndesmotic instability assessed by intraoperative stress tests could be detected in the case of injury to two or more ligaments [18,37,43]. Therefore, we can assume that only PITFL injuries do not lead to clinical syndesmotic instability, and conservative management can be considered for type 3 PMF if syndesmotic instability is not detected in intraoperative stress tests. The concept that in pure PITFL avulsion fractures (type 3 PMF), only the restoration of the ankle’s lateral and medial components is enough to obtain stability and lead to satisfactory outcomes has been supported by several studies [11,12,14,19,21]. However, if syndesmosis instability is suspected, it may indicate PITFL avulsion accompanied by medial ligament injury or other associated damage. In such cases, careful evaluation is necessary to determine the need for syndesmosis fixation, medial ligament repair, and appropriate management of any additional injuries.

In terms of complications, the surgical fixation group showed significantly more complications. For the type of fixation, the plate fixation method showed significantly more complications of metal irritations and hallux flexion deficits.

The concept of PMF treatment is rapidly and widely changing toward a more aggressive approach; however, it remains controversial [11,12,25,26,28,29]. According to our results, the optimal indication and technique for operative PMF treatment could be selected based on the specific injury mechanism; notably, direct reduction and plate osteosynthesis might not be beneficial for certain morphological PMF subtypes. However, further biomechanical understanding of this challenging injury and clinical evidence with a larger sample and a longer follow-up period is needed.

This study has certain limitations. Since this was a retrospective study, data were collected by a medical record review and were not strictly controlled; hence, insufficient information regarding sural nerve injury or plate/screw fixation indication may have affected the outcomes as a confounder.

The patients who additionally underwent PMF fixation in the prone position following unsatisfactory reduction, where the PMF was unfixed in the supine position, were assigned to the fixation group, which implies a level of selection bias in this retrospective study. We did not measure the amount of articular involvement of the PMF or fracture fragment size, which is classically considered the main prognostic factor for patient outcomes. However, this “dogma” appears to have shifted due to increasing evidence demonstrating that PMF morphology and injury mechanisms rather than fragment size are more predictive [1,3,11,12,14,16,17,26,46]. We did not evaluate the presence of osteochondral injuries detected by either preoperative MRI or intraoperative joint inspection; such injuries are more frequent in type 2 PMF than in other subtypes and are associated with an increased risk of poor functional outcomes [47]. In addition, we could not use a comprehensive complication rating system, such as the Clavien-Dindo classification. How each complication was managed needs to be evaluated to calculate the scoring system. Instead, we completed the number of each complication as a statistical comparison of each group. If we could use a systematic complication scoring system, it could have more clinically meaningful findings. Another limitation of this study is the absence of multivariate regression analysis to identify predictors of clinical and radiological outcomes. Given that our study conducted multiple independent analyses based on Haraguchi’s classification, we chose not to perform regression analysis to avoid adding further complexity to an already intricate study design. We believe that conducting detailed subgroup analyses based on the presence of ICF could have provided more meaningful statistical results. Clinically, the decision to perform surgery is often significantly influenced by the presence of ICF. In this study, we have just described this aspect retrospectively based on observed data. For future research, we plan to include analyses of surgical outcomes according to fracture types and surgical decisions influenced by the presence of ICF, which we believe will lead to more meaningful conclusions.

## 5. Conclusions

In conclusion, different treatment strategies are required for each PMF morphological subtype. Despite the current concept favoring the operative treatment, including the direct approach and plate fixation, there is insufficient evidence to suggest that this technique guarantees favorable outcomes for all the PMF subtypes. According to the results of this study, in type 1 fractures, if there is no ICF, and also the reduction of the posterior malleolar fracture is satisfactory on intraoperative fluoroscopy, and the deltoid ligament is either intact or successfully repaired to ensure medial ligament stability, it appears that secure syndesmosis fixation alone can achieve surgical outcomes comparable to those in the group with direct fixation of the posterior malleolar fracture. In type 2 fractures, the presence of ICF is common, and the comminuted nature of these fractures makes it challenging to achieve proper reduction through non-surgical methods. As a result, surgical reduction and fixation appear to be associated with superior surgical outcomes compared to non-surgical approaches. In type 3 fractures, surgical fixation of the posterior malleolar fragment is unnecessary, and the evaluation and stabilization of syndesmosis instability should be considered the primary determining factors.

## Figures and Tables

**Table 1:** Comparison between the PMF non-fixation and fixation groups within the type 1 PMF.

	Non-Fixation Group	Fixation Group	*p*
	(N = 183)	(N = 54)	
Age ^a^ (year) ^†^	45.8 ± 14.9	43.3 ± 17.5	0.295
Sex			0.648
Female	93 (50.8%)	30 (55.6%)	
Male	90 (49.2%)	24 (44.4%)	
Side of fracture			0.522
Left	111 (60.7%)	36 (66.7%)	
Right	72 (39.3%)	18 (33.3%)	
F/U duration (month) ^†^	48.8 ± 8.2	47.3 ± 4.3	0.400
Fracture type			0.173
Deltoid ligament rupture	42 (23.0%)	18 (33.3%)	
Medial malleolar fracture	141 (77.0%)	36 (66.7%)	
Weber’s type			0.097
B	150 (82.0%)	38 (70.4%)	
C	33 (18.0%)	16 (29.6%)	
L-H type			0.222
PAB	6 (3.3%)	4 (7.4%)	
PER	30 (16.4%)	12 (22.2%)	
SER	147 (80.3%)	38 (70.4%)	
Injury mechanism			0.753
slip	111 (60.7%)	35 (64.8%)	
sports	33 (18.0%)	10 (18.5%)	
TA	39 (21.3%)	9 (16.7%)	
BMI (Kg/m^2^) ^†^	24.9 ± 3.4	25.1 ± 1.6	0.658
Intercalary fragment			**<0.001**
no	147 (80.3%)	18 (33.3%)	
yes	36 (19.7%)	36 (66.7%)	
Quality of reduction by CT			0.269
good	153 (83.6%)	45 (83.3%)	
fair	18 (9.8%)	8 (14.8%)	
poor	12 (6.6%)	1 (1.9%)	
Step-off			0.931
no	177 (96.7%)	53 (98.1%)	
yes	6 (3.3%)	1 (1.9%)	
PTOA			0.725
no	168 (91.8%)	51 (94.4%)	
yes	15 (8.2%)	3 (5.6%)	
Syndesmosis instability			**<0.001**
no	153 (83.6%)	30 (55.6%)	
yes	30 (16.4%)	24 (44.4%)	
Syndesmosis stabilization			0.434
no	153 (83.6%)	42 (77.8%)	
yes	30 (16.4%)	12 (22.2%)	
AOFAS ^†^	91.7 ± 5.4	92.4 ± 2.6	0.166
OMAS ^†^	90.2 ± 5.9	90.6 ± 2.5	0.417
FAOS (total) ^†‡^	89.2 ± 6.2	90.4 ± 2.2	0.079
VAS	1.54 ± 1.07	1.46 ± 0.61	0.771
Postoperativecomplication			**0.008**
no	149 (81.4%)	34 (63.0%)	
yes	34 (18.6%)	20 (37.0%)	

^a^ Age at surgery. Bolds are significant values. The values are given as the number of patients with the percentage in parenthesis unless noted otherwise. ^†^ The values are given as the mean and the standard deviation. ^‡^ All data of FAOS are represented as scores changed based on 100 points. F/U, follow-up; L-H type, Lauge-Hansen type; PAB, pronation-abduction injury; PER, pronation-external rotation injury; SER, supination-external rotation injury; TA, traffic accident; BMI, body mass index; CT, Computed Tomography; PTOA, post-traumatic osteoarthritis; AOFAS, American Orthopaedic Foot and Ankle Society hindfoot Score; VAS, visual analog scale; OMAS, Olerud and Molander Score; FAOS, Foot and Ankle Outcome Score.

**Table 2:** Comparison between the PMF non-fixation and fixation groups within the type 2 PMF.

	Non-Fixation Group	Fixation Group	*p*
	(N = 92)	(N = 107)	
Age ^a^ (year) ^†^	46.0 ± 15.3	46.5 ± 12.1	0.804
Sex			0.489
Female	62 (67.4%)	78 (72.9%)	
Male	30 (32.6%)	29 (27.1%)	
Side of fracture			0.579
Left	62 (67.4%)	67 (62.6%)	
Right	30 (32.6%)	40 (37.4%)	
F/U duration (month) ^†^	52.7 ± 7.9	55.9 ± 16.9	0.085
Fracture type			0.172
Deltoid ligament rupture	0 (0.0%)	4 (3.7%)	
Medial malleolar fracture	92 (100.0%)	103 (96.3%)	
Weber’s type			0.906
B	55 (59.8%)	62 (57.9%)	
C	37 (40.2%)	45 (42.1%)	
L-H type			0.774
PAB	5 (5.4%)	4 (3.7%)	
PER	37 (40.2%)	47 (43.9%)	
SER	50 (54.3%)	56 (52.3%)	
Injury mechanism			0.557
slip	62 (67.4%)	71 (66.4%)	
sports	5 (5.4%)	10 (9.3%)	
TA	25 (27.2%)	26 (24.3%)	
BMI (Kg/m^2^) ^†^	24.6 ± 2.3	24.6 ± 3.0	0.983
Intercalary fragment			**0.020**
no	25 (27.2%)	14 (13.1%)	
yes	67 (72.8%)	93 (86.9%)	
Quality of reduction by CT			**<0.001**
good	5 (5.4%)	65 (60.7%)	
fair	35 (38.0%)	34 (31.8%)	
poor	52 (56.5%)	8 (7.5%)	
Step-off			**<0.001**
no	30 (32.6%)	96 (89.7%)	
yes	62 (67.4%)	11 (10.3%)	
PTOA			**<0.001**
no	41 (44.6%)	89 (83.2%)	
yes	51 (55.4%)	18 (16.8%)	
Syndesmosis instability			1.000
no	47 (51.1%)	55 (51.4%)	
yes	45 (48.9%)	52 (48.6%)	
Syndesmosis stabilization			**<0.001**
no	41 (44.6%)	85 (79.4%)	
yes	51 (55.4%)	22 (20.6%)	
AOFAS ^†^	72.1 ± 8.0	91.0 ± 4.3	**<0.001**
OMAS ^†^	69.8 ± 6.6	90.2 ± 4.3	**<0.001**
FAOS (total) ^†‡^	69.3 ± 11.3	89.3 ± 5.0	**<0.001**
VAS	3.5 ± 1.4	1.7 ± 1.1	**<0.001**
Postoperative complication			0.366
no	60 (65.2%)	62 (57.9%)	
yes	32 (34.8%)	45 (42.1%)	

^a^ Age at surgery. Bolds are significant values. The values are given as the number of patients with the percentage in parenthesis unless noted otherwise. ^†^ The values are given as the mean and the standard deviation. ^‡^ All data of FAOS are represented as scores changed based on 100 points. F/U, follow-up; L-H type, Lauge-Hansen type; PAB, pronation-abduction injury; PER, pronation-external rotation injury; SER, supination-external rotation injury; TA, traffic accident; BMI, body mass index; CT, Computed Tomography; PTOA, post-traumatic osteoarthritis; AOFAS, American Orthopaedic Foot and Ankle Society hindfoot Score; VAS, visual analog scale; OMAS, Olerud and Molander Score; FAOS, Foot and Ankle Outcome Score.

**Table 3:** Regression analysis results on key factors influencing surgical fixation of posterior malleolar fragments for predicting worse treatment outcomes. (Bolds are significant values).

	Odd Ratio (95% Confidence Interval)	*p*
Age	1.016 (0.985 to 1.048)	0.324
Sex	1.349 (0.564 to 3.223)	0.501
Body mass index	0.940 (0.756 to 1.169)	0.577
Post-traumatic osteoarthritis	6.212 (1.275 to 30.270)	**0.024**
Step-off	18.945 (3.942 to 91.044)	**<0.001**

**Table 4:** Comparison between the syndesmosis stabilization and non-stabilization group within the Type 3 PMF.

	Non-Stabilization Group	Stabilization Group	*p*
	(N = 21)	(N = 15)	
Age ^a^ (year) ^†^	46.3 ± 15.1	41.4 ± 5.9	0.239
Sex			0.845
Female	15 (71.4%)	12 (80.0%)	
Male	6 (28.6%)	3 (20.0%)	
Side of fracture			0.457
Left	15 (71.4%)	12 (80.0%)	
Right	6 (28.6%)	3 (20.0%)	
F/U duration (month) ^†^	44.7 ± 19.4	49.0 ± 12.8	0.376
Fracture type			0.845
Deltoid ligament rupture	6 (28.6%)	3 (20.0%)	
Medial malleolar fracture	15 (71.4%)	12 (80.0%)	
Weber’s type			1.000
B	18 (85.7%)	12 (80.0%)	
C	3 (14.3%)	3 (20.0%)	
L-H type			1.000
PER	3 (14.3%)	3 (20.0%)	
SER	18 (85.7%)	12 (80.0%)	
Injury mechanism			0.845
slip	15 (71.4%)	12 (80.0%)	
TA	6 (28.6%)	3 (20.0%)	
BMI (Kg/m^2^) ^†^	26.1 ± 0.3	24.6 ± 2.8	0.465
Intercalary fragment			1.000
no	18 (85.7%)	12 (80.0%)	
yes	3 (14.3%)	3 (20.0%)	
Quality of reduction by CT			0.892
good	17 (81.0%)	11 (73.3%)	
fair	4 (19.0%)	4 (26.7%)	
PTOA			0.126
no	21 (100.0%)	12 (80.0%)	
yes	0 (0.0%)	3 (20.0%)	
Syndesmosis instability			**<0.001**
no	21 (100.0%)	0 (0.0%)	
yes	0 (0.0%)	15 (100.0%)	
AOFAS ^†^	88.0 ± 7.82	92.0 ± 2.54	0.162
OMAS ^†^	87.0 ± 7.7	90.4 ± 1.4	0.060
FAOS (total) ^†‡^	87.5 ± 6.1	91.3 ± 1.8	0.263
VAS	1.86 ± 1.28	1.40 ± 0.83	0.147
Postoperative complication			1.000
no	15 (71.4%)	11 (73.3%)	
yes	6 (28.6%)	4 (26.7%)	

^a^ Age at surgery. Bolds are significant values. The values are given as the number of patients with the percentage in parenthesis unless noted otherwise. ^†^ The values are given as the mean and the standard deviation. ^‡^ All data of FAOS are represented as scores changed based on 100 points. F/U, follow-up; L-H type, Lauge-Hansen type; PAB, pronation-abduction injury; PER, pronation-external rotation injury; SER, supination-external rotation injury; TA, traffic accident; BMI, body mass index; CT, Computed Tomography; PTOA, post-traumatic osteoarthritis; AOFAS, American Orthopaedic Foot and Ankle Society hindfoot Score; VAS, visual analog scale; OMAS, Olerud and Molander Score; FAOS, Foot and Ankle Outcome Score.

**Table 5:** Comparison between the screw fixation and plate fixation groups.

	Screw Group	Plate Group	*p*
	(N = 134)	(N = 27)	
Haraguchi’s classification			**0.042**
H1	50 (37.3%)	4 (14.8%)	
H2	84 (62.7%)	23 (85.2%)	
Age ^a^ (year) ^†^	44.5 ± 13.2	50.1 ± 18.0	0.129
Sex			0.128
Female	86 (64.2%)	22 (81.5%)	
Male	48 (35.8%)	5 (18.5%)	
Side of fracture			1.000
Left	86 (64.2%)	17 (63.0%)	
Right	48 (35.8%)	10 (37.0%)	
F/U duration (month) ^†^	52.1 ± 14.9	55.6 ± 13.7	0.260
Fracture type			1.000
Deltoid ligament rupture	18 (13.4%)	4 (14.8%)	
Medial malleolar fracture	116 (86.6%)	23 (85.2%)	
Weber’s type			0.324
B	86 (64.2%)	14 (51.9%)	
C	48 (35.8%)	13 (48.1%)	
L-H type			0.679
PAB	6 (4.5%)	2 (7.4%)	
PER	48 (35.8%)	11 (40.7%)	
SER	80 (59.7%)	14 (51.9%)	
Injury mechanism			0.015
slip	82 (61.2%)	24 (88.9%)	
sports	21 (15.7%)	0 (0.0%)	
TA	31 (23.1%)	3 (11.1%)	
BMI (Kg/m^2^) ^†^	24.9 ± 2.8	23.4 ± 2.7	0.156
Intercalary fragment			**0.010**
no	32 (23.9%)	0 (0.0%)	
yes	102 (76.1%)	27 (100.0%)	
Quality of reduction by CT			0.093
good	87 (64.9%)	23 (85.2%)	
fair	38 (28.4%)	4 (14.8%)	
poor	9 (6.7%)	0 (0.0%)	
Step-off			0.224
no	122 (91.0%)	27 (100.0%)	
yes	12 (9.0%)	0 (0.0%)	
PTOA			0.522
no	115 (85.8%)	25 (92.6%)	
yes	19 (14.2%)	2 (7.4%)	
Syndesmosis instability			0.073
no	66 (49.3%)	19 (70.4%)	
yes	68 (50.7%)	8 (29.6%)	
Syndesmosis stabilization			0.534
no	104 (77.6%)	23 (85.2%)	
yes	30 (22.4%)	4 (14.8%)	
AOFAS ^†^	91.4 ± 4.0	91.8 ± 3.2	0.597
OMAS ^†^	90.3 ± 4.0	90.6 ± 3.0	0.717
FAOS (total) ^†‡^	89.9 ± 4.1	88.9 ± 4.3	0.219
VAS	1.55 ± 1.02	1.78 ± 0.64	0.082
Postoperative complication			0.797
no	81 (60.4%)	15 (55.6%)	
yes	53 (39.6%)	12 (44.4%)	

^a^ Age at surgery. Bolds are significant values. The values are given as the number of patients with the percentage in parenthesis unless noted otherwise. ^†^ The values are given as the mean and the standard deviation. ^‡^ All data of FAOS are represented as scores changed based on 100 points. F/U, follow-up; L-H type, Lauge-Hansen type; PAB, pronation-abduction injury; PER, pronation-external rotation injury; SER, supination-external rotation injury; TA, traffic accident; BMI, body mass index; CT, Computed Tomography; PTOA, post-traumatic osteoarthritis; AOFAS, American Orthopaedic Foot and Ankle Society hindfoot Score; VAS, visual analog scale; OMAS, Olerud and Molander Score; FAOS, Foot and Ankle Outcome Score.

**Table 6:** Comparison of the postoperative complications between the PMF non-fixation and fixation group.

	Non-Fixation Group	Fixation Group	*p*
	(N = 311)	(N = 161)	
Postoperative complication			**<0.001**
no	235 (75.6%)	96 (59.6%)	
yes	76 (24.4%)	65 (40.4%)	
Superficial wound infection			0.163
no	278 (89.4%)	136 (84.5%)	
yes	33 (10.6%)	25 (15.5%)	
Deep wound infection			**<0.001**
no	309 (99.4%)	150 (93.2%)	
yes	2 (0.6%)	11 (6.8%)	
SPN			0.116
no	301 (96.8%)	150 (93.2%)	
yes	10 (3.2%)	11 (6.8%)	
Metal irritation			0.751
no	288 (92.6%)	147 (91.3%)	
yes	23 (7.4%)	14 (8.7%)	
Etc			**<0.001**
no	304 (97.7%)	151 (93.8%)	
hallux flexion deficit	0 (0.0%)	8 (5.0%)	
hypertrophic scar	0 (0.0%)	2 (1.2%)	
LOM	3 (1.0%)	0 (0.0%)	
peroneal nerve palsy	1 (0.3%)	0 (0.0%)	
T-F synostosis	3 (1.0%)	0 (0.0%)	

Bolds are significant values. The values are given as the number of patients, with the percentage in parentheses. SPN, superficial nerve injury; LOM, limitation of range of motion; T-F synostosis, tibio-fibular synostosis.

**Table 7:** Comparison of the complications between the screw fixation and plate fixation group.

	Screw Group	Plate Group	*p*
	(N = 134)	(N = 27)	
Postoperative complication			0.797
no	81 (60.4%)	15 (55.6%)	
yes	53 (39.6%)	12 (44.4%)	
Superficial wound infection			0.324
no	111 (82.8%)	25 (92.6%)	
yes	23 (17.2%)	2 (7.4%)	
Deep wound infection			1.000
no	125 (93.3%)	25 (92.6%)	
yes	9 (6.7%)	2 (7.4%)	
SPN			1.000
no	125 (93.3%)	25 (92.6%)	
yes	9 (6.7%)	2 (7.4%)	
Metal irritation			**<0.001**
no	128 (95.5%)	19 (70.4%)	
yes	6 (4.5%)	8 (29.6%)	
Etc			**0.001**
no	130 (97.0%)	21 (77.8%)	
hallux flexion deficit	3 (2.2%)	5 (18.5%)	
hypertrophic scar	1 (0.7%)	1 (3.7%)	

Bolds are significant values. The values are given as the number of patients, with the percentage in parentheses. SPN, superficial nerve injury.

## Data Availability

All data used in this study are included within the article, and additional data are available at reasonable request from the authors.
